# Prevalence of inappropriate medication use in residential long-term care facilities for the elderly: A systematic review

**DOI:** 10.1080/13814788.2017.1288211

**Published:** 2017-03-08

**Authors:** Hannelore Storms, Kristel Marquet, Bert Aertgeerts, Neree Claes

**Affiliations:** ^a^ Faculty of Medicine and Life Sciences, Hasselt UniversityHasseltBelgium; ^b^ Quality and Safety Department, Jessa HospitalHasseltBelgium; ^c^ Department of Public Health and Primary Care, Academic Centre for General Practice, KU Leuven; Centre for EBM-CEBAMLeuvenBelgium; ^d^ Antwerp Management School, Faculty Leadership, Health Care ManagementAntwerpBelgium

**Keywords:** General practice/family medicine, general, pharmacotherapy, geriatrics, multimorbidity, quality of care, systematic reviews and meta-analyses, research, methodology

## Abstract

**Background:** Multi-morbidity and polypharmacy of the elderly population enhances the probability of elderly in residential long-term care facilities experiencing inappropriate medication use.

**Objectives:** The aim is to systematically review literature to assess the prevalence of inappropriate medication use in residential long-term care facilities for the elderly.

**Methods:** Databases (MEDLINE, EMBASE) were searched for literature from 2004 to 2016 to identify studies examining inappropriate medication use in residential long-term care facilities for the elderly. Studies were eligible when relying on Beers criteria, STOPP, START, PRISCUS list, ACOVE, BEDNURS or MAI instruments. Inappropriate medication use was defined by the criteria of these seven instruments.

**Results:** Twenty-one studies met inclusion criteria. Seventeen studies relied on a version of Beers criteria with prevalence ranging between 18.5% and 82.6% (median 46.5%) residents experiencing inappropriate medication use. A smaller range, from 21.3% to 63.0% (median 35.1%), was reported when considering solely the 10 studies that used Beers criteria updated in 2003. Prevalence varied from 23.7% to 79.8% (median 61.1%) in seven studies relying on STOPP. START and ACOVE were relied on in respectively four (prevalence: 30.5–74.0%) and two studies (prevalence: 28.9–58.0%); PRISCUS, BEDNURS and MAI were all used in one study each.

**Conclusions:** Beers criteria of 2003 and STOPP were most frequently used to determine inappropriate medication use in residential long-term care facilities. Prevalence of inappropriate medication use strongly varied, despite similarities in research design and assessment with identical instrument(s).

KEY MESSAGES

- In residential long-term care facilities for the elderly, healthcare professionals find monitoring inappropriate medication use important.
- Beers criteria and STOPP rules were most frequently used.
- The high prevalence of inappropriate medication use indicates the need for medication monitoring systems.

## Introduction

Monitoring inappropriate medication use in an elderly population is crucial because of their frailty due to multi-morbidity and associated polypharmacy. Consequently, the risk of drug-drug and drug–disease interactions as well as errors being made due to complex medication regimens increases. These errors in medication management can affect patients’ health outcomes as inappropriate medication use is associated with higher hospitalization rates and mortality in older patients [1,2]. Cahir et al., for instance, found 42% community-dwelling elderly to be experiencing inappropriate medication use with being twice as likely to experience an adverse drug event when taking at least two potentially inappropriate drugs [3]. Unfortunately, no studies have yet been carried out to support a similar hypothesis in a residential care setting [4]. However, a prevalence study by O’Sullivan et al. illustrates the likelihood of the elderly to experience inappropriate medication: with more than half of the 732 participating nursing home residents experiencing potentially inappropriate prescribing [5].

It is apparent that little research on preventable inappropriate medication use has been carried out in residential long-term care facilities, as an important setting for older people. To illustrate, in 2014, 506.8 million people were living in the European Union and almost 94 million of them were aged 65 years and over. EUROSTAT data from 2014 on living conditions calculated almost 4% of people aged 65 years old and over living in Germany, France or Denmark to be residing in long-term care facilities (not hospitals); in the Netherlands and the Nordic countries (Sweden, Norway, Finland) this is 5% and in Belgium this is 9% [6]. Moreover, the turnover of nurses, which characteristically is relatively high in these institutionalized care settings, as well as the absence of on-site physicians or pharmacists in some of them, might result in difficulties with medication follow-up [7,8]. The scarcity of research in residential long-term care facilities is in stark contrast with the extensive research on the potential harm of drug therapies targeting an elderly population that has been carried out in hospital settings [1]. To illustrate, Hug et al. reported 120 out of 180 preventable adverse drug events of which respectively 8.9% and 6.7% could be prevented by drug-age checking and by drug-specific guidelines [9].

The aim of this systematic review is to determine the exposure of residents in long-term care facilities for the elderly to inappropriate medication use expressed as the prevalence of inappropriate medication use.

## Methods

### Search strategy

**Figure 1.**
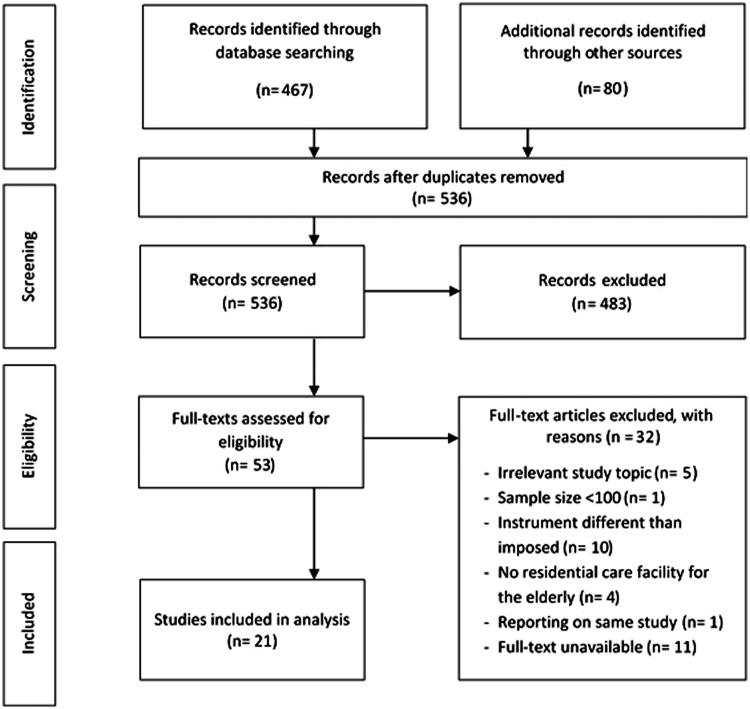
Flow diagram of study-selection process

Electronic databases (EMBASE, MEDLINE) were searched from January 2004 to March 2016 to identify studies examining the prevalence of inappropriate medication use in residential long-term care facilities – HP.2 Classification of Health Care Providers (ICHA-HP) [10] – for the elderly. Research articles and conference papers written in English, Dutch, French and German relying on minimum one of seven instruments frequently used to assess inappropriate medication use (Beers criteria, STOPP, START, PRISCUS list, ACOVE, BEDNURS and MAI [11–21]) were considered (Table 1) [22–24]. Keywords ‘medication errors’ and ‘adverse drug event’ in the setting ‘nursing home’ were also included in the search strategy. Additional studies of interest were searched in reference lists of included articles (Figure 1). A full electronic search strategy is provided in a supplemental file.

**Table 1.** Characteristics of researched instruments.

	Appropriateness of medication use	Drug-drug interactions	Drug–disease interactions	Overtreatment	Therapeutic duplications	Underuse
Beers criteria	+		+			
STOPP	+	+	+		+	
START						+
PRISCUS	+					
ACOVE	+					+
BEDNURS	+	+		+		+
MAI	+	+	+		+	

STOPP: screening tool of older persons’ potentially inappropriate prescriptions; START: screening tool to alert right treatment; ACOVE: assessing care of vulnerable elders; BEDNURS: Bergen District Nursing Home; MAI: medication appropriateness index.

### Selection of studies: inclusion and exclusion criteria

Two independent researchers (HS, KM) screened titles, abstracts and full texts in correspondence with inclusion criteria. When abstracts were not available, the full text, if available, was consulted. Research articles were included when inappropriate medication use was examined, in a setting of residential care for the elderly and when at least one of seven studied instruments was used for assessment.

Exclusion criteria were: less than 100 residents participating in the study, irrelevant study design or topic, and research in which only medication administration errors were examined.

### Quality assessment of studies

A quality assessment of included studies was carried out using critical appraisal skills programme (CASP) tools. Allocation concealment, as well as random sequence generation, were irrelevant in generating potential bias because only baseline data were found eligible for this review. Researchers could not be blinded to outcome because assessment of inappropriate medication use implied the use of sets of criteria on data. Because of non-randomization, included studies can be subjected to sampling bias (number of nursing homes; representativeness of sample; geographical area of participating nursing homes). Information on the loss of participants was examined to address attrition bias. Transparency in data reporting was assessed to determine reporting bias – data clearly stated; potential bias being addressed; critical reflection on data (limitations and strengths). The quality of studies was categorized low, medium or high: a representative setting, clearly stated data (proportion versus total number), and a rigorous description of methodology were prevailing domains in the quality assessment of included studies.

### Data extraction and analysis

Two researchers (HS, KM) independently extracted data using a pre-defined extraction form (Microsoft Excel). Discordances were resolved by consensus. Research with incomplete data was excluded from analysis because of risk on bias. In case of studies with multiple settings, only data of residential long-term care facilities for the elderly were considered. Regarding intervention studies, data of the initial review of medication (before intervention) were retained. Studies comparing intervention groups to control groups were regarded as generating two separate populations (baseline data of both groups were analysed). Despite overlap between criteria of predefined instruments, the prevalence assessed with different instruments could not be compared. The prevalence of inappropriate medication use was expressed as the percentage of residents experiencing inappropriate medication use. For studies relying on MAI, inappropriate medication use was set out as a sum score with standard deviation. Additionally, descriptive data (number, mean and standard deviation) on drug use of residents were reported. No meta-analysis could be realized because of heterogeneity in data resulting from varying study designs, length of studies and resources for data gathering.

## Results

Twenty-one out of 536 studies identified, met inclusion criteria. The main reasons for exclusion are described in the flow diagram of the study selection process (Figure 1).

### Characteristics

Most studies were carried out in Europe (Table 2) [5,22,25–31]. In seventeen studies (81%), researchers assessed inappropriate medication use relying on criteria developed by Beers et al., with the majority (10 studies) relying on the criteria updated in 2003 (Table 2) [11–14]. STOPP-criteria are used in seven (33%) studies, being the second most referred to [15,18]. Three studies assessed the prevalence of inappropriate medication use relying on Beers criteria updated in 2012 (Table 2) [11]. Only one study used the implicit tool ‘MAI’ [16,19,28]. Some researchers relied on additional (national) tools: these were often inspired by the Beers criteria [22,25,29,32]. Seven studies used several instruments, in particular, Verrue et al., and Elseviers et al. [22,28].

**Table 2.** Characteristics of included studies (*n* = 21).

						Per cent of residents experiencing inappropriate medication use						Sum score	
Author, year, country [Ref.]	Set of criteria^a^	Residential long-term care facilities *n*	Residents *n* (% women)	Age Mean (SD)	Medication use *n*; *mean*; (SD)	Beers criteria	STOPP	START	PRISCUS	ACOVE	BEDNURS	MAI	Quality assessment
King et al., 2007, Australia [39]	B1991	15	998 (71)	83.6 (NA)	NA	18.5							Low
Niwata et al., 2006, Japan [43]	B2003 (ID-CD^b^]	17	1669 (74.7)	84.5 (NA)	NA	21.3							Medium
Varallo et al., 2012, Brazil [35]	B2003 (ID)	1	120 (NA)	NA	116	29.2							Low
Hosia-Randell et al., 2008, Finland [29]	B2003 (ID)	20	1987 (80.7)	83.7 (7.7)	*7.9*	34.9							Medium
Stafford et al., 2011, Australia [32]	B2003 (ID-CD^b^]	41	2345 (75.5)	87 (NA)	17529	35.3							Low
Barnett et al., 2011, UK [25]	B2003 (ID)	NA	4557 (72.3)	84.5 (7.5)	NA	37.1							High
Perri et al., 2005, USA [37]	B1997	15	1117 (81.6)	84.6 (8.08)	NA	46.5							Medium
Ruggiero et al., 2010, Italy [26]	B2003 (ID-CD^b^]	31	1716 (71.7)	83.64 (8.06)	NA	48.0							High
Lau et al., 2004, USA [42]	B1991 + B1997	NA	3372 (73.8)	NA	NA	50.3							High
Hwang et al., 2015, South Korea [38]	B2012 (ID-CD^b^]	20	529 (23.3)	80.8 (NA)	NA	58.2							Medium
Pinto et al., 2013, Brazil [34]	B2003 + B2012 (ID-CD^b^]	5	151 (62.9)	76.69 (10.97)	*3.31* (1.8)	63.0							Medium
Mamun et al., 2004, Singapore [41]	B1997	3	454 (66.7)	80 (NA)	2048	70.0							Low
Vieira de Lima et al., 2013, Brazil [36]	B2012 (ID-CD^b^]	6	261 (57.5)	NA	1452	82.6							Medium
Verrue et al., 2012, Belgium [28) Intervention and control group	B1997 STOPP START ACOVE MAI	2	148 (70.6)	82.7 (NA)	NA	48.9	53.8	30.5		28.9		**11.2**	Medium
O'Sullivan et al., 2013, Ireland [5]	B2003 (ID-CD^b^) STOPP	14	732 (70.2)	83.9 (7.7)	8325	53.6	70.8						High
Chen et al., 2012, Malaysia [33]	B2003 (ID-CD^b^) STOPP	4	211 (60.7)	77.7 (7.0)	*4.7* (2.8)	32.7	23.7						Medium
Elseviers et al., 2014, Belgium [22]	B2003 (ID) PRISCUS ACOVE BEDNURS	76	1730 (78.1)	84.8 (NA)	NA	27.0			NA	58.0	56.0		Low
García-Gollarte, 2012, Spain [31]	STOPP START	6	100 (80)	84.7 (7.5)	6.49 (2.86)		79.0	74.0					Low
García-Gollarte, 2014, Spain [30) Intervention group	STOPP START	36	516 (74.0)	84.24 (14.6)	*8.25* (3.39)		66.8	55.1					Low
García-Gollarte, 2014, Spain [30) Control group	STOPP START	36	502 (72.1)	84.5 (10.4)	*7.89* (3.27)		62.4	48.6					Low
Ryan et al., 2013, Ireland [27]	STOPP START	7	313 (74.4)	84.4 (7.5)	2555		59.8	42.2					Medium
Lao et al., 2013 China [40]	STOPP	1	114 (66.7)	86.6 (8.4)	114		46.5						Medium

^a^ Beers criteria year of creation.

^b^ Explicit distinction between independent from disease and considering disease.

ID, independent of disease; CD: considering disease; NA, no information available; SD: standard deviation.

The number of participants in the reviewed studies ranged from 100 to 4557 residents (Table 2). Eligibility for participation mostly depended on meeting an age requirement, overall being aged 65 years or more. Furthermore, reviewed studies excluded residents who required palliative care, who’s data were incomplete or if they were transferred or died during the study period. Data on inappropriate medication use were gathered through medical records [5,27,30,31,32–38], medication charts and databases [5,22,25,26,28,29,39–43].

### Prevalence of inappropriate medication use

The prevalence of inappropriate medication use varied from 18.5% to 82.6% (median 46.5%) when relying on Beers criteria in general (‘B1991,’ ‘B1997,’ ‘B2003’ and ‘B2012’ in Table 2). Prevalence ranged from 21.3% to 63.0% (median 35.1%) when inappropriate medication use was assessed solely relying on the complete criteria list of Beers 2003 (‘B2003,’ both criteria independent of disease ‘ID’ and criteria considering disease ‘CD’) (Table 2). Reported prevalence of inappropriate medication use was 63.0% and 82.6% in studies relying on the 2012 update of the Beers criteria (‘B2012’) [34,36]. Studies based on STOPP, reported a prevalence of 23.7% to 79.8% (median 61.1%); prevalence ranged between 30.5% and 74.0% (median 48.6%) for research based on START (Table 2). In Table 3, drug classes most prevalent inappropriately used were listed per study: these were often benzodiazepines.

**Table 3.** Drug classes most prevalent inappropriately used as proportion inappropriate medication use (IMs) or proportion residents.

	Drug classes most prevalent inappropriately used (% of IMs)				
Author, year [Ref.]	Beers criteria	STOPP^a^	START	ACOVE	BEDNURS
Varallo et al., 2012 [35]	Diazepam (28.9)				
Barnett et al., 2011 [25]	Ferrous sulphate >325 mg/d (13.9)				
Ruggiero et al., 2010 [26]	Ticlopidine (8.1)				
Vieira de Lima et al., 2013 [36]	Antipsychotics (27.8)				
Verrue et al., 2012 [28) Intervention and control group	Benzodiazepine use in patients with depression (31.7)	Long-term use of benzodiazepines (17.9)	Acetylsalicylic acid for diabetics (24.1)	Acetylsalicylic acid for diabetics (24.1)	
O'Sullivan et al., 2013 [5]	ID Chlordiazepoxide and diazepam (29.2) CD Falls/syncope with short-to-intermediate-acting benzodiazepines or TCAs (35.2)	Benzodiazepines in individuals with a history of recurrent falls (15.4)			
Chen et al., 2012 [33]	ID Nifedipine, short acting (46.8) CD Syncope or falls: Short-to intermediate-acting benzodiazepine and tricyclic antidepressants (2.5)	A fall in the past three months + first generation antihistamine (daily or as needed basis) (23.4)			
García-Gollarte, 2012 [31]		The use of a proton-pump inhibitor (PPI) without a clear indication [52]	Not using vitamin D and calcium in patients with osteoporosis [34]		
Ryan et al., 2013 [27]		Benzodiazepines (25.8)	Aspirin (19.6)		
Lao et al., 2013 [40]		Benzodiazepines in patients with a fall (12.6)			
King and Roberts, 2007 [39]	Diazepam (10.4)				
Niwata et al., 2006 [43]	Ticlopidine (6.3)				
Hosia-Randell et al., 2008 [29]	Short-acting benzodiazepines (13.9)				
Stafford et al., 2011 [32]	Benzodiazepines (16.7)				
Perri et al., 2005 [37]	Propoxyphene (14.4)				
Pinto and Malaquias, 2013 [34]	Risperidone (20.53)				
Mamun et al., 2004 [41]	Antihistamines (85.7)				
Elseviers et al., 2014 [22]	Digoxin [7]			Heart failure without treatment with a beta-blocker [23]	Psychotropic medication combined use of ATC classes N05 (psycholeptics) + N06 (psychoanaleptics) [32]

^a^ STOPP-criterion ‘duplicate classes’ disregarded.

STOPP: screening tool of older persons’ potentially inappropriate prescriptions; START: screening tool to alert right treatment; ACOVE: assessing care of vulnerable elders; BEDNURS: Bergen District Nursing Home; CD: considering disease; ID: independent of disease; NA: no information available.

## Discussion

### Main findings

Most studies that relied on the instruments most frequently used – Beers criteria or STOPP – were, despite their origin, carried out in Europe: respectively eight in European countries and two in the USA versus five in Europe and zero in the USA. Divergence in scope as well as differently expressed results, caused heterogeneity in presented data. Consequently, a meta-analysis was hampered.

### Prevalence of inappropriate medication use and assessment criteria

This review suggests a strongly varying prevalence of inappropriate medication use in institutionalized care settings for the elderly. The prevalence did not seem to correlate with the extent of the assessment of inappropriate medication use: studies relying on more extensive criteria, did not report a higher prevalence of inappropriate medication use. However, consistent with previous research, the prevalence of inappropriate medication use detected by STOPP was, except for one study [33], higher than assessed with Beers criteria [5,28,33]. Studies with similar characteristics relying on identical instruments could not be compared, not even within the same (continental) region. The latter can be attributable to country specific market regulations, resulting in medication being unavailable and therefore variations in the applicability of identical instruments.

### Prevalence of inappropriate medication use and polypharmacy

In studies relying on STOPP, with a similar sample size, a similar prevalence was found (53.8% and 46.5% versus 66.8% and 62.4%) [28,30,40]. The same can be stated about studies based on the 2003 Beers criteria with similarities in number of participating residents, average drug use, as well as (not) taking into account residents’ diseases (‘ID’) (48.9% and 34.9%) [22,29]. Limited data hampered any further statement on contributing or causal factors.

In the five studies based on Beers 2003 with condition-dependent criteria (B2003 ‘CD’), the proportion of residents experiencing inappropriate medication use (53.6%) was the highest in studies with residents taking the highest number of drugs [5,26,32,33]. An analogous trend – a correlation between residents using a high number of drugs and a high prevalence of inappropriate medication use – could be distinguished in research based on Beers criteria updated in 2012 [34,36]. The reviewed studies suggest a correlation between inappropriate medication use and polypharmacy in residential long-term care facilities [5,26,27,29,32,33,36,38,41–43], in analogy with both hospital as home-care settings [1,44,45]. If there is a higher risk on inappropriate medication use due to polypharmacy, as suggested in this review, it is valuable to monitor inappropriate medication use in residential long-term care facilities’ populations [26,37,43,46,47]. A European study across eight countries, concluded 49.7% of 4023 residents to be experiencing polypharmacy (taking five to nine drugs) and 24.3% residents experiencing excessive polypharmacy (taking 10 or more drugs) [48].

### Implications for policy and research

Variations in or incomparability of prevalence reported in this review can be partly explained by geographical variations in medication being unavailable, therefore affecting data. Because of its repercussions on generalizability, prevalence studies will be more valuable when data are generated based on actual medication use rather than on available medication only. In light of globalization, problems and advantages of implicit or explicit guidelines to assess the inappropriate use of medication need to be weighed up as patients might have access to – and therefore take – medication that is not authorized on the local, national, continental market. An approach to bypass the obstacle of medication not being on the market is the use of a more generic tool, for instance an instrument like the ‘appropriate medication for older people tool’ [49]. A downside of the latter is that healthcare professionals are required to have sufficient knowledge about medication and its characteristics (drug-drug interactions, side effects).

Future policies should promote systematically executed medication reviews to make them standard practice in residential long-term care facilities. Ideally, when assessments are carried out more frequently, researchers as well as healthcare professionals would become accustomed to it and might even feel the need to use these measurements as quality indicators and to benchmark. Consequently, this could be beneficial for methodological quality of studies, for instance by being transparent on prevalence, by providing information on the loss of participants and by encouraging complete medication history taking.

An accurate medication overview is a prerequisite determining if medication is clinically indicated, particularly for elderly people with polypharmacy [50,51]. To inform healthcare professionals about all medication taken by the resident, it is crucial that it is regularly updated. Moreover, this updated medication overview should allow healthcare professionals to detect inappropriate medication use quickly. Moreover, by incorporating evidence-based guidelines in clinical decision support systems, the prescriber can be alerted of possible hazardous drug (-disease) combinations [23,50,52,53].

### Strengths and limitations

Studies included in this review generated heterogeneous data because of diversity in study design; study period and how inappropriate medication use is expressed (about medication being used or as people subjected to inappropriate medication use). Based on preliminary, narrow search strategies – with restrictions on study design and the use of one (or two) instrument(s) assessing inappropriate medication use – a broad search strategy was chosen to gather sufficient data. It was decided: to research only two databases because no additional, relevant studies were found searching others (e.g. CINAHL); and to research the seven instruments based on geographical origin (USA, Europe) and their merit resulting in frequent use and therefore the potential to generate studies that might be comparable to some extent [4]. Consequently, studies reporting on prevalence of inappropriate medication use using other assessment tools are not included. The chosen study period allowed detection of studies relying both on older instruments and relatively new ones. However, the broadened search strategy also resulted in a varying methodological quality of eligible studies. Quality assessment of included studies most frequently consisted of determining sampling, attrition and reporting bias. In numerous studies, palliative care patients were excluded. Because for this group of people medication use can be expected to relate strongly to their complex condition, including them would probably result in a higher prevalence of inappropriate medication use. However, exclusion might be justified as there might not be a medicinal alternative to manage these patients’ conditions. Studies lacked information on loss of participants, disregarding the (only) mentioning of exclusion because of ‘incomplete’ data. Several studies required recalculations, because of non-transparent data.

In this review, inappropriate medication use among residents was set out as point prevalence. Weighing (heterogeneous) data would have improved data visualization and would have corrected data spreading. However, in our opinion, given varying scope and methodologies of included studies, analysis based on weighed data would have had a relatively small impact on the main findings.

To the best of our knowledge, this is the first review to gain insight into the extent of inappropriate medication use in residential long-term care facilities for the elderly, explicitly considering various instruments for assessment. Despite the restrictions, findings of this review suggest an awareness of the importance to monitor inappropriate medication use.

## Conclusion

Inappropriate medication use is broadly defined as inappropriate medication use by the criteria described by the instruments set out to be researched (Beers criteria, STOPP, START, PRISCUS list, ACOVE, BEDNURS, MAI) [11–21]. Prevalence of inappropriate medication use was most often assessed relying on the Beers criteria updated in 2003 and STOPP [14,15,18]. Heterogeneity in data hampered meta-analysis, limiting statements on the prevalence of inappropriate medication use: prevalence of inappropriate medication use strongly varies, even among studies with similar characteristics. Despite varying quality, numerous studies assessed inappropriate medication use, suggesting an awareness to monitor inappropriate medication use in residential long-term care facilities in the elderly.

## Supplementary Material

EMBASE SEARCH STRATEGY

PRISMA 2009 Checklist
