# Supplementary material for: Prevalence of inappropriate medication use in residential long-term care facilities for the elderly: A systematic review
Source: Eur J Gen Pract. 2017 Mar 8;23(1):69–77. doi: 10.1080/13814788.2017.1288211 (PMC5774291; doi:10.1080/13814788.2017.1288211)
Supplement: EMBASE SEARCH STRATEGY [file igen_a_1288211_sm0608.pdf]

EMBASE SEARCH STRATEGY

'nursing home'/exp OR 'nursing home' AND ('adverse drug event'/exp OR 'adverse drug event' AND [2004-2016]/py OR ('medication errors'/exp OR 'medication errors' AND [2004-2016]/py) AND 'beers criteria' AND [2004-2016]/py OR (acove AND [2004-2016]/py) OR (bednurs AND [2004-2016]/py) OR ('medication appropriateness index'/exp OR 'medication appropriateness index' AND [2004-2016]/py) OR (stopp AND [2004-2016]/py) OR (start AND [2004-2016]/py)) AND ([dutch]/lim OR [english]/lim OR [french]/lim OR [german]/lim) AND [humans]/lim AND [2004-2016]/py AND ('article'/it OR 'article in press'/it
